# Digital Photoplethysmography for Assessment of Arterial Stiffness: Repeatability and Comparison with Applanation Tonometry

**DOI:** 10.1371/journal.pone.0135659

**Published:** 2015-08-20

**Authors:** Emma von Wowern, Gerd Östling, Peter M. Nilsson, Per Olofsson

**Affiliations:** 1 Department of Obstetrics and Gynecology, Institution of Clinical Sciences, Skåne University Hospital, Lund University, Malmö, Sweden; 2 Medical Research Unit, Department of Internal Medicine, Institution of Clinical Sciences, Skåne University Hospital, Lund University, Malmö, Sweden; Vanderbilt University Medical Center, UNITED STATES

## Abstract

**Introduction:**

Arterial stiffness is an independent risk factor for cardiovascular morbidity and can be assessed by applanation tonometry by measuring pulse wave velocity (PWV) and augmentation index (AIX) by pressure pulse wave analysis (PWA). As an inexpensive and operator independent alternative, photoelectric plethysmography (PPG) has been introduced with analysis of the digital volume pulse wave (DPA) and its second derivatives of wave reflections.

**Objective:**

The objective was to investigate the repeatability of arterial stiffness parameters measured by digital pulse wave analysis (DPA) and the associations to applanation tonometry parameters.

**Methods and Results:**

112 pregnant and non-pregnant individuals of different ages and genders were examined with SphygmoCor arterial wall tonometry and Meridian DPA finger photoplethysmography. Coefficients of repeatability, Bland-Altman plots, intraclass correlation coefficients and correlations to heart rate (HR) and body height were calculated for DPA variables, and the DPA variables were compared to tonometry variables left ventricular ejection time (LVET), PWV and AIX. No DPA variable showed any systematic measurement error or excellent repeatability, but dicrotic index (DI), dicrotic dilatation index (DDI), cardiac ejection elasticity index (EEI), aging index (AI) and second derivatives of the crude pulse wave curve, *b*/*a* and *e*/*a*, showed good repeatability. Overall, the correlations to AIX were better than to PWV, with correlations coefficients >0.70 for EEI, AI and *b*/*a*. Considering the level of repeatability and the correlations to tonometry, the overall best DPA parameters were EEI, AI and *b*/*a*. The two pansystolic time parameters, ejection time compensated (ETc) by DPA and LVET by tonometry, showed a significant but weak correlation.

**Conclusion:**

For estimation of the LV function, ETc, EEI and *b*/*a* are suitable, for large artery stiffness EEI, and for small arteries DI and DDI. The only global parameter, AI, showed a high repeatability and the overall best correlations with AIX and PWV.

## Introduction

Increased arterial wall stiffness secondary to endothelial dysfunction and arteriosclerosis is a risk factor for cardiovascular morbidity [1,2]. Arterial stiffness can be determined non-invasively by recording the pulse wave (PW) velocity (PWV) and by analyzing the aortic pressure PW contour properties. Regarding PW properties, the relation between the forward (primary) PW and the PW reflection in distal arteries (tidal PW) is commonly expressed as augmentation index (AIX), i.e. the amplification of the forward PW curve caused by the overlap of the reflected PW. At an increased arterial stiffness, the forward PW travels faster in the arteries, the reflected PW returns earlier to the point of measurement, and the reflected PW augments the forward PW. The PWV is correlated to age, blood pressure (BP) and arteriosclerosis and has a better predictive value for cardiovascular disease than traditional risk markers [3,4]. AIX is influenced by both large artery stiffness and properties in the PW-reflecting peripheral arterial tree [5–7] and is associated with cardiovascular risk, although it is less established than the PWV as a risk factor for cardiovascular morbidity [8–10].

Arterial stiffness parameters can be determined by applanation tonometry methods, where pressure sensors are placed on superficial arteries, e.g. the carotid, radial and femoral arteries. The carotid-to-femoral PWV is regarded the gold standard to assess arterial stiffness [2]. Although pressure PW analysis shows benefits in predicting cardiovascular morbidity [1,10], the impact in clinical practice is small because the tonometry method is cumbersome with a need for highly specialized operators. As an alternative, digital volume PW analysis (DPA) with photoelectric plethysmography (PPG) has been introduced. This technology is inexpensive, easy to use and not operator dependent, which makes it accessible and suitable for clinical practice [11]. Millasseau et al. [12] have in parallel recordings showed that there are close correlations between the contours and characteristics of the peripheral arterial pressure PW and the digital volume PW.

A crude PPG recording allows analysis of the amplitude of the PW but not much of details of the contour itself. A mathematical ‘remodelling’ of the PW components with second derivatives enables analyses of the anacrotic and dicrotic notches of the crude PW curve. However, a full understanding of the second derivative wave details is still lacking, though several DPA parameters correlate to vascular pathology [4,11,13–19]. The aim of the present cross-sectional non-interventional study was to investigate the repeatability of arterial wall elasticity parameters measured by the Meridian DPA photoplethysmograph, and the associations between the DPA parameters and the cardiac left ventricular (LV) ejection time (ET), AIX and PWV recorded by a tonometry SphygmoCor apparatus in pregnant women and in non-pregnant individuals of different ages and gender.

## Material and Methods

The study was performed at the Medical Research Unit, Department of Internal Medicine, Skåne University Hospital in Malmö, Sweden. It was approved by the Regional Research Ethics Committee in Lund (Dnr. 532/2006) and all participants gave their oral and written informed consent.

Volunteers recruited for the study were men and women age 64 years and above recruited from an ongoing screening study at the Medical Research Unit, and non-pregnant women up to 64 years and pregnant women recruited from the Department of Obstetrics and Gynecology (Table 1). The physical condition, smoking and medications of the volunteers were disregarded, and no volunteer was recruited because of treatment at the hospital. All participants were asked to avoid alcohol for at least 12 h and large meals, coffee, tea and nicotine for at least 3 h prior to the examinations. Medications should be taken as prescribed. The temperature in the examination room was 22±1°C. After at least 5 min of rest in the supine position and before the PW recordings started, the left brachial artery BP was recorded twice with a one minute interval with an automatic device (Omron Intellisense model M8 RC, Omron Healthcare, Hoofdorp, The Netherlands). Three tonometry measurements and two DPA recordings were performed in series during the same séance. Only high quality recordings according to the apparatuses’ own quality securing system were accepted.

**Table 1:** Demographic characteristics of the study group.

	Non-pregnant women		Pregnant women		Men		All	
	n = 73		n = 14		n = 25		n = 112	
	Median (range)	Mean ± SD	Median (range)	Mean ± SD	Median (range)	Mean ± SD	Median (range)	Mean ± SD
**Age** (years)	54 (21–84)	53 ± 17	31 (25–45)	33 ± 5	71 (64–83)	72 ± 6	64 (21–84)	55 ± 18
**Height** (cm)	166 (146–180)	166 ± 7	169 (156–180)	169 ± 6	176 (161–190)	176 ± 7	168 (146–190)	168 ± 8
**BMI** (kg/m^2^)	25.3 (17.6–41.7)	25.8 ± 4.9	25.9 (20.6–32.4)	26.4 ± 2.9	27.1 (18.4–35.8)	27.4 ± 3.6	25.9 (17.6–41.7)	26.2 ± 4.5
**SBP** (mmHg)	121 (99–250)	124 ± 21	116 (99–128)	114 ± 9	124 (104–185)	128 ± 17	121 (99–250)	124 ± 19
**DBP** (mmHg)	72 (58–102)	73 ± 8	74 (65–83)	74 ± 5	74 (63–88)	74 ± 6	73 (58–102)	73 ± 7
**Heart rate** (bpm)	65 (51–100)	66 ± 11	70 (60–92)	74 ± 11	61 (44–93)	62 ± 12	64 (44–100)	66 ± 12

BMI, body mass index; SBP, systolic blood pressure; DBP, diastolic blood pressure

### Arterial pulse wave analysis with applanation tonometry

Davies & Struther [20] have described the applanation tonometry method with assessment of the pressure PW. In our study, a SphygmoCor device was used (CvMS version 9, AtCor Medical, West Ryde, NSW, Australia; www.atcormedical.com). The apparatus and the method have been described in a white paper by Skinner et al. [21]. The SphygmoCor calculates the central aortic pressure PW by a transformation algorithm from the pressure PW recorded noninvasively from a peripheral artery. Thus, the central AIX is calculated as the ratio between the augmentation pressure and the total pulse pressure, expressed in percent. Studies have reported a good correlation between the AIX determined from measured and derived aortic pressure PWs (R 0.75, *P*<0.001) [12,22]. The arterial PWs were recorded by a hand-held pencil tonometer placed on the carotid, radial and femoral arteries. The AIX, derived from the radial artery tonometry recordings, is a measure of the reflected PW and the additional load to the LV; thus, AIX is dependent on cardiac cycle length and heart rate (HR), PWV and the reflected wave amplification [20]. The forward pressure PW amplitude increases when recorded more distal from the heart and the amplification is positively correlated to the HR [21], but due to an alteration in the relative timing of the reflected pressure PW, the AIX is inversely related to HR [23]. For this reason, the SphygmoCor automatically corrects the AIX to a fictive HR of 75 bpm, denoted AIX@75 in this paper.

The PWV were recorded with the tonometer placed on the carotid and femoral arteries. By ECG synchronization and measurements of the distances between the recording points with a vernier calliper, the heart-to-carotid and heart-to-femoral PWVs could be calculated and expressed in m/s.

**Fig 1:**
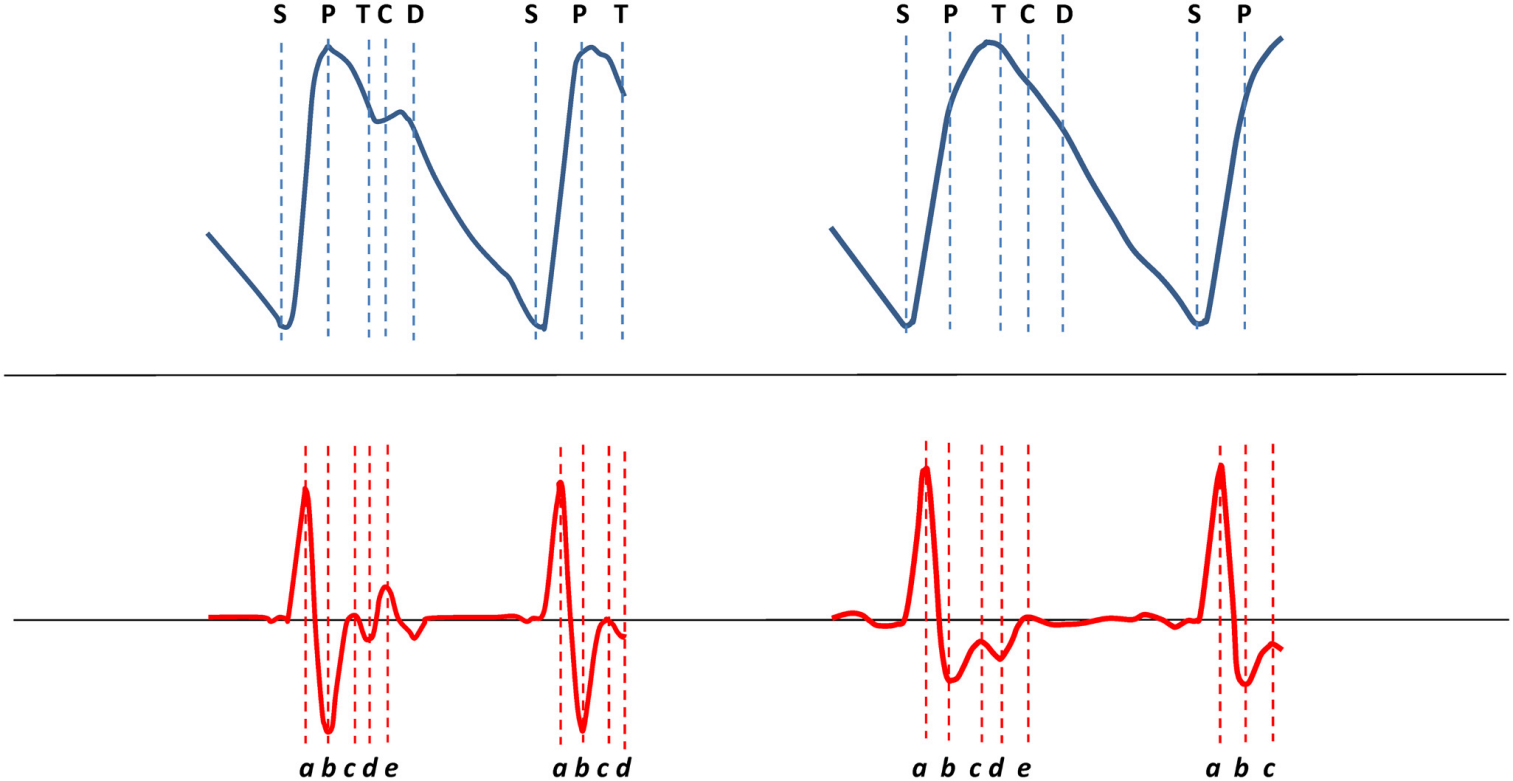
Digital photoplethysmograms and acceleration photoplethysmograms. Authentic digital photoplethysmograms (upper panels) of a 33-year old healthy woman (left panel, heart rate 65 bpm, aging index -0.87) and a 66-year old healthy man (right panel, heart rate 61, aging index -0.09). S = starting point of systole; P = peak of percussion wave; T = tidal wave; C = incisura wave; D = dicrotic wave. Note the absence of a distinct incisura and dicrotic notch in the right plethysmogram, and differences in negative amplitudes of *b* and *d* peaks in the acceleration plethysmograms (lower panels).

The LVET (in the SphygmoCor called LV ejection duration), i.e. the pansystolic time expressed in milliseconds (ms), is the time elapsing from the start of the LV contraction (corresponding to point S in the DPA curve, Fig 1) till closure of the aortic valves (corresponding to the incisura wave, point C, in the DPA curve, Fig 1) [21]. A sharp incisura (point C) is not always present in the radial pressure PW, but with analysis of higher derivatives of the PW contour the incisura is located with reasonable accuracy [21]. Thus, the systolic time is estimated from the peripheral pressure PW after transformation to the derived aortic pulse.

### Digital pulse wave analysis with photoplethysmography

We used the terminology for digital PPG proposed by Elgendi [24]. Millasseau and colleagues [25] have described the physiological background to the contour analysis of the PPG PW measured in a finger. In our study a Meridian DPA was used (Salcor AB, Uppsala, Sweden; www.meridian.co.kr). A clip with a light-emitting diode (LED) on the nail side and a light receiving photodiode on the opposite side was placed on the left index finger. The DPA works by emitting infrared light at a wavelength of 940 nm through the finger to the photodiode and on its way the light is absorbed relative to the oxyhemoglobin content (red cell density) in the tissue (and the expansion of the vascular and tissue volume); blood volume changes in the finger can thus be recorded by measuring the non-absorbed light and a PPG PW be formed. A crude PPG recording allows analysis of the amplitude of the PW but not of the contour itself. A mathematical ‘remodelling’ of the PW components by second derivation of the PW curve recorded by PPG was therefore proposed by Ozawa [26]. Thus, a graph of a function with a positive second derivative curves upwards, and a graph of a function with a negative second derivative curves downwards (Fig 1). This ‘acceleration PPG’ (APG) allows for detailed evaluations of the curvatures of the PPG waveform, i.e. the accelerative and decelerative phases of the curve contour, and a more accurate recognition of the inflection points on the original photoplethysmogram, i.e. the anacrotic and dicrotic notches (Fig 1). The dicrotic notch corresponds to closure of the aortic valve and its identification (by derivatives of the crude PPG) is critical for analysing the systolic time interval [27] in both DPA recordings and applanation tonometry.

The Meridian DPA automatically analyses HR and 16 different PW parameters. A recording takes 70 seconds to perform. For accurate analysis at least 80% of the PWs must be recognized and a prerequisite for good PW recognition is a good pulse height (PH). Poor PH can be caused by nail polish, acrylic nails or cold hands. When needed, nail polish was removed and the hand warmed by a heating pad.

### Photoplethysmographic variables

The physiological explanations of the different PPG and APG indices were obtained from the manufacturer’s manual (Meridian DPA, Meridian Co., Ltd., Korea), by personal email communication with Mr. Saint Myeong and Mr. Steve Kim at the Meridian Company, and from the literature. To facilitate the readability of the article, references to personal communication are not indicated in the text. Much research related to the APG has been performed in Japan and South Korea and much is published in the native languages, which restricts our possibility to go back to the original sources of publication. The reference intervals for normal values provided by the manufacturer were not used in our study.

The capital letters S, P, T, C, and D of the PPG refer to curve points as shown in the upper panels of Fig 1, and the small letters *a* through *e* of the APG refer to curve points as shown in the lower panels. The waveform elements are: S = starting point of arterial pulse wave. The aortic valve opens and blood is ejected from the LV; P = percussion (forward) wave. The LV blood ejection is transmitted by the elastic arterial wall. A higher peak means stronger LV ejection and higher compliance of large arteries [28]; T = tidal (reflected) wave. The T-wave is reflected from the small arteries. Higher peak means contraction and stiffness of small arteries [28]; C = incisura wave. C marks the end-point of systole, prior to aortic valve closing. A less drop from PH means larger resistance (arterial contraction and tension) [28]; D = dicrotic wave. A reflective oscillatory wave occurring when the blood crash into the aortic valve by pressure in the aorta.

#### Pulse height (PH)

In general, the pulse amplitude relates to the LV ejection performance, cardiac stroke volume and large artery distensibility [28–30], though the PPG pulse amplitude relates also to blood flow in small finger arteries. A high PH can indicate a high BP, hyperthyroidism, fever, anemia, excessive blood volume, atherosclerosis, anxiety, and exercise. A high PH can also be seen in well-tuned athletes. A low PH can indicate peripheral vasoconstriction, low blood pressure, hypovolemia/dehydration, hypothyroidism, or increased peripheral resistance. Cold hands and Raynaud’s phenomenon cause a low PH. Dehydration is characterized by a low PH and low dicrotic elasticity index (DEI, see below), i.e. a low peripheral artery compliance due to a centralized circulation. Because of potential environmental and/or conditional influences on PH, PH was not analyzed as an outcome variable in this study.

#### Ejection time compensated (ETc)

ETc = (time from S to C)/√HR_bps_ expressed in milliseconds (ms), where HR_bps_ denotes heart beats per second (bps). ETc corresponds to the LVET, i.e. the time from onset of the systolic upstroke limb to the closure of the aortic valve (points S to C in Fig 1). The ET varies inversely with the HR and directly with stroke volume [31] and ETc is therefore adjusted to a HR of 60 bpm (beats/second = bpm/60) in the Meridian DPA, reading the calculation equation as ETc = (S-C time)/√(HR/60); this is a non-linear HR correction equation, originally presented by Bazett in 1920 [32]. The LVET is increased in aortic valve disease and decreased in LV muscle failure, with decreased preload, and with positive and negative inotropic agents [33]. A prolonged ETc could indicate heart insufficiency with impaired cardiac output and aortic valve problems like stenosis. An abnormally short ETc could indicate hyperthyroidism, diastolic hypertension, and a small LV.

#### Elasticity index (EI)

EI = P/T. EI is a measure of LV ejection capacity and arterial stiffness and compliance (elasticity) of large arteries. A low EI indicates vasoconstriction, arteriosclerosis or LV ejection insufficiency; a high EI indicates arterial dilation or increased ejection power of the LV.

#### Cardiac ejection elasticity index (EEI)

EEI = (P/T) × (-b/a). EEI is an index for LV ejection capacity and compliance/elasticity of large arteries. A low EEI indicates vasoconstriction, arteriosclerosis or LV ejection insufficiency; a high EEI indicates vasodilation of large arteries, anemia, increased ejection power, hyperthyroidism or congested heart failure.

#### Angle of descent index or dicrotic index (DI)

DI = C/PH. DI represents the peripheral circulation (capillaries and veins) and indicates peripheral resistance; DI decreases at vasodilation.

#### Dicrotic dilatation index (DDI)

DDI = (PH-C)/PH = 1-C/PH = 1-DI. DDI is an index for elasticity in small arteries. A low DDI indicates vasoconstriction or arteriosclerosis, a high DDI indicates vasodilation.

#### Dicrotic elasticity index (DEI)

DEI = (D-D’)/C, where the distance D-D’ is the overshoot of the dicrotic wave over a straight line from C to S. DEI is an index for compliance/elasticity of small arteries/arteriole or venous blood flow. A low DEI indicates arteriolar constriction, arteriosclerosis or environmental effects such as cold, caffein, nicotine, or emotions with increased sympathetic tone; a high DEI indicates arteriolar dilation, low sympathetic tone, vasodilatory drugs, or venous insufficiency.

By the second derivative, four separate systolic wave peaks (denoted *a* to *d*) and one diastolic peak (denoted *e*) are obtained on the APG (Fig 1). The *a* and *b* wave peaks occur in the early systolic phase and waves *c* and *d* in the late systolic phase. The *e* wave represents the dictrotic notch and corresponds to the aortic valve closure [11]. The height from the baseline of each wave can be measured and ratios between them calculated (*b*/*a*, *c*/*a*, *d*/*a*, and *e*/*a*). In 1998 Takazawa and coworkers [14] published their classical study, where the APG parameters recorded in the index fingertip of the left hand were validated against invasive measurements of the ascending aortic pressure during manipulations of the vascular arterial tone with angiotensin (vasoconstriction) and nitroglycerin (vasodilation), and the APG parameters were compared with demographic characteristics in an epidemiological study. Studies have demonstrated that the APG parameters are closely associated with aging and risk factors for arteriosclerotic vascular disease [13–15], but it is still unclear which one is clinically most informative [11].

**Ratio *b*/*a*** relates to the rising systolic slope from S to P, i.e. the early systolic phase. The influence of the reflected PW on the *a* and *b* waves is negligible [14,16,17]; hence, the *b*/*a* ratio is an indicator of the LV ejection capacity and large artery compliance/elasticity. Young persons have low absolute ratio values, i.e. a long negative *b* peak resulting in negative absolute *b*/*a* values, and the negative *b* peak shortens by aging [14–18]. The *b*/*a* ratio correlates positively to the Framingham cardiovascular risk score [17]. A low ratio (= high negative ratio) indicates high compliance/elasticity, a high ratio indicates low elasticity, vasoconstriction, and arteriosclerosis. *b*/*a* was not affected by vasoactive drugs in the study by Takazawa et al. [14].

**Ratio *c*/*a*** reflects the late systolic phase, just before the T wave. The *c*/*a* ratio reflects arterial stiffness and decreases with age [14,18], i.e. the *c* peak moves downwards and *c*/*a* becomes more negative with aging.

**Ratio *d*/*a*** represents the T wave. The *d* wave mainly reflects the intensity of the tidal PW from small peripheral arteries [17]. A longer negative *d* peak develops by advancing age, i.e. the *d*/*a* ratio decreases, indicating arterial stiffness [14,16–18]. The *d*/*a* ratio correlates negatively to the Framingham score [17]. An increasing negative value represents constriction and/or stiffness in small arteries. The *d*/*a* ratio is closely related to the augmentation of BP in the aorta by wave reflection [16] and it is a useful index to assess LV afterload and effects of vasoactive agents [14]. In the study by Takazawa et al. [14], the *d*/*a* ratio was markedly affected by the vasoactive agents without significant effects on the *b*/*a*, *c*/*a*, and *e*/*a* ratios.

**Ratio *e*/*a*** represents the early diastolic phase, C and D waves, during relaxation of the heart. The *e*/*a* ratio decreases with age [14,18].

#### Aging index (AI)

AI = (*b*/*a*)–(*c*/*a*)–(*d*/*a*)–(*e*/*a*) = (*b*-*c*-*d*-*e*)/*a*. AI represents the ‘vascular age’, i.e. arterial stiffness. The lower value, i.e. the more negative, the better; AI increases with age and arteriosclerosis [4,14,16]. AI should not be mistaken for AIX, measured by the applanation tonometry methods.

The intervals *a*-*b*, *a*-*c*, *a*-*d*, and *a*-*e* indicate the time elapsing between each wave peak (crest time). Since the *d* wave mainly reflects the intensity of the reflected PW from small peripheral arteries [17], one can expect that the time for the reflected tidal wave to travel is proportional to the height of the individual [19], and a prolonged *a*–*d* time might indicate vasodilation or low vascular tone.

### Statistical analyses

We applied the recommendations by Bartlett & Frost [34] and Vaz et al. [35] to calculate the repeatability of the PPG and APG variables. In this context the *agreement* between repeated measurements is a characteristic of the method or instrument and is not dependent of characteristics of the population investigated, whereas the *reliability* depends on both the magnitude of measurement errors (bias) and the heterogeneity in the population. Since a DPA measurement is to just press a button, there was no need to take inter-observer variations into consideration. *Reproducibility* refers to measurements during changing conditions [34] and was not evaluated in this study.

*Agreement* quantifies how close measurements made on the same subject are under identical conditions over a short period of time [34]. We described agreement between the two DPA measurements with Bland-Altman plots [36,37]. The upper and lower limits of agreement (LoA) were calculated as the mean difference between measurements (delta) ± 1.96 SD and contain 95% of the paired measurements. The upper LoA minus the mean difference between measurements represents the coefficient of repeatability (CoR). A Bland-Altman plot visually demonstrates whether the bias varies systematically over the range of measurements.

*Reliability* relates to the magnitude of the measurement error and was expressed as the intraclass correlation coefficient (ICC) of model 2 for individual ratings [38], calculated with a statistics tool box provided online (http://department.obg.cuhk.edu.hk/index.asp?scr=1920). The ICC takes dimensionless values between zero and one [34,35], where the value of (1 –ICC) relates to the errors in the measurement process. An ICC >0.75 indicates good reliability, 0.50–0.75 moderate reliability, and <0.50 a poor reliability [38].

The mean value of multiple measurements was used in the comparisons with demographic characteristics and between the equipments. Associations between continuous variables were tested with Spearman’s rank correlation coefficient (rho); to reveal linear correlations, simple and second order of polynomial linear regression analyses with Pearson’s correlation coefficient (r) were performed. A two-sided *p* value of <0.05 was regarded significant.

As stated by Jang et al. [39], linear regression equations can be used for adjustments of time-related PPG parameters. In cases a linear regression analysis showed a significant association between a DPA parameter and HR and/or body height, the DPA parameter values were adjusted accordingly to a fictive fixed HR of 75 bpm (denoted variable@75), a fictive fixed height of 170 cm (denoted variable@170), or both to a fixed HR and fixed height (variable@75@170), as calculated with the obtained simple or second order regression equation.

The adjustments of DPA variables were performed in a hierarchical order, where adjustments for HR were first performed and then each adjusted variable@75 was compared with body height in new linear regression analyses and the DPA variable@75 adjusted accordingly to a variable@75@170. The adjusted variables were then compared with the SphygmoCor tonometry variables PWV and AIX@75.

The DPA variable ETc and the tonometer variable LVET were compared separately because these variables are the only ones directly corresponding to each other, measuring the pansystolic time; although the ETc is automatically adjusted to a HR of 60 bpm by Bazett’s equation, the LVET values were adjusted to a HR of 60 bpm (LVET@60) by the obtained linear regression equation (see Discussion).

The StatView version 5.0.1 (SAS Institute, Cary, NC, USA) and MedCalc (MedCalc Software, Mariakerke, Belgium) computer softwares were used for the statistical calculations.

## Results

### Agreement and reliability of DPA variables

**Fig 2:**
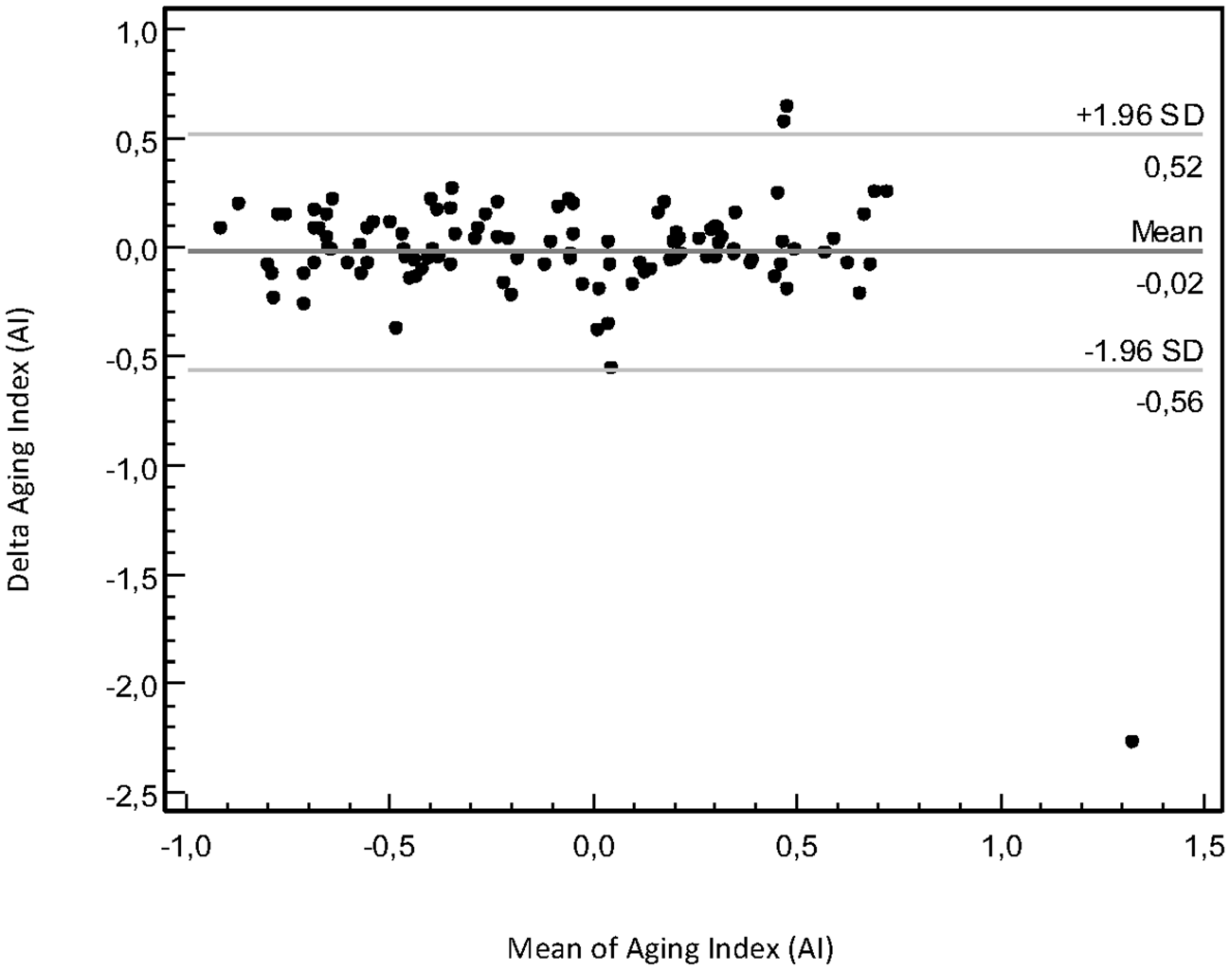
Repeatability of aging index. Bland-Altman plot of two consecutive measurements of aging index (AI) by the Meridian DPA apparatus, depicting the relationship of difference of measurements (Delta AI) and mean value of measurements.

The agreement and reliability measures of repeated DPA measurements are shown in Table 2. The Bland-Altman plots showed for no DPA variable any systematic skewness along the range of measurement values (plot shown only for AI, Fig 2). The mean difference of measurements was not considerably distant from zero for any variable, though the LoAs were wide and the CoRs were high for some variables (Table 2). The LoA and CoR are sensitive to outliers and outliers may be omitted from analysis [36], and when visually examining the Bland-Altman plots three individuals (see footnote to Table 2) were identified with outlying values for several variables; omitting these persons resulted in considerably better results for especially DEI, AI, *c*/*a* and *d*/*a* (Table 2).

**Table 2:** Measures of agreement and reliability between two repeated measurements of digital pulse analysis (DPA) variables in 112 individuals. For explanation of DPA variables, see text.

DPA variable	1^st^ measurement		2^nd^ measurement		Mean of measurements	1^st^ vs. 2^nd^ measurement, simple linear regression analysis		Agreement: Bland-Altman analysis						Reliability
	Mean	SD	Mean	SD		*p*	r	-1.96 SD^a^	Mean ΔDPA	+1.96 SD^b^	CoR	Mean ΔDPA^c^	CoR^c^	ICC
**ETc**	341.86	36.41	342.92	30.21	344.73	< 0.0001	0.47	-69.54	-1.29	66.97	68.25	-2.4	67.1	0.46
**EI**	0.97	0.26	0.96	0.25	0.97	< 0.0001	0.72	-0.39	0.015	0.42	0.38	0.00	0.35	0.72
**DI**	0.74	0.15	0.75	0.13	0.74	< 0.0001	0.80	-0.18	-0.010	0.16	0.17	0.002	0.14	0.80
**DDI**	0.26	0.15	0.25	0.13	0.26	< 0.0001	0.81	-0.15	0.015	0.18	0.17	0.008	0.14	0.81
**EEI**	0.49	0.29	0.49	0.29	0.50	< 0.0001	0.82	-0.34	0.004	0.35	0.35	0.00	0.28	0.82
**DEI**	0.08	0.23	0.06	0.09	0.060	< 0.0001	0.42	-0.39	0.030	0.45	0.41	0.01	0.17	0.27
**AI**	-0.10	0.45	-0.07	0.51	-0.093	< 0.0001	0.84	-0.56	-0.020	0.52	0.54	0.00	0.34	0.84
***b*/*a***	-0.48	0.17	-0.48	0.18	-0.48	< 0.0001	0.83	-0.20	0.002	0.20	0.20	-0.01	0.18	0.84
***c*/*a***	-0.19	0.14	-0.20	0.15	-0.20	< 0.0001	0.63	-0.23	0.008	0.25	0.24	0.00	0.15	0.63
***d*/*a***	-0.31	0.17	-0.33	0.24	-0.31	< 0.0001	0.53	-0.39	0.013	0.41	0.40	-0.01	0.21	0.53
***e*/*a***	0.12	0.09	0.12	0.08	0.12	< 0.0001	0.88	-0.088	-0.003	0.082	0.084	0.00	0.073	0.87
***a*-*b***	94.36	27.79	93.88	26.25	94.70	< 0.0001	0.54	-50.39	0.64	51.67	51.48	0.60	51.7	0.55
***a*-*c***	173.72	28.76	177.20	29.80	176.66	0.0082	0.25	-73.24	-3.30	66.64	70.20	-3.2	69.8	0.26
***a*-*d***	233.50	35.15	238.22	28.68	236.82	< 0.0001	0.40	-72.53	-3.90	64.73	69.19	-4.9	67.0	0.40
***a*-*e***	331.23	37.09	331.82	40.54	334.12	< 0.0001	0.71	-59.24	-0.87	57.50	58.44	-2.1	53.1	0.72

ΔDPA, difference between measurements; CoR, coefficient of repeatability; ICC, intraclass correlation coefficient

^a)^ 95% lower limit of agreement (-1.96 × SD of ΔDPA).

^b)^ 95% upper limit of agreement (+1.96 × SD of ΔDPA).

^c)^ After exclusion of three outliers with ΔDPA values ≥ ± 4SD of mean ΔDPA (old age in two, low heart rate in two, and low pulse height in one).

A good reliability was found for DI, DDI, EEI, AI, *b*/*a*, and *e*/*a* (ICC 0.80–0.87), a moderate reliability for EI, *c*/*a*, *d*/*a*, *a*-*b*, and *a*-*e* (ICC 0.53–0.72), whereas a poor reliability was found for ETc, DEI, *a*-*c*, and *a*-*d* (ICC 0.26–0.46).

### Associations between DPA variables and heart rate and body height

There was no significant correlation between body height and HR (Spearman’s rank correlation, *p* = 0.56; simple linear regression, *p* = 0.98) and these variables were therefore considered to be independent of each other.

Table 3 shows the correlations between DPA variables and the HR and body height, respectively, and the HR-adjusted DPA variables vs. body height. There were significant correlations between the DPA variables and HR for all variables except *a*-*c* (Spearman’s rank correlation, *p* = 0.058) (Table 3); the relations were significant and linear for all variables, among which *d*/*a* correlated with HR at a second order of polynomial regression analysis only.

**Table 3:** Correlation between DPA variables and heart rate and body height, respectively, in 112 individuals. DPA@75 denotes adjustment of the variable to a heart rate of 75 bpm.

DPA variables	DPA vs. heart rate				DPA vs. body height				DPA@75 vs. body height			
	Spearman’s rank correlation		Simple linear regression		Spearman’s rank correlation		Simple linear regression		Spearman’s rank correlation		Simple linear regression	
	*p*	*rho*	*p*	*r*	*p*	*rho*	*p*	*r*	*p*	*rho*	*p*	*r*
**EI**	<0.0001	0.56	<0.0001	0.60	0.27	-	0.28	-	0.16	-	0.19	-
**DI**	<0.0001	-0.64	<0.0001	-0.65	0.90	-	0.84	-	1.0	-	0.81	-
**DDI**	<0.0001	0.66	<0.0001	0.66	0.87	-	0.93	-	0.92	-	0.75	-
**EEI**	<0.0001	0.44	<0.0001	0.46	0.27	-	0.42	-	0.22	-	0.28	-
**DEI**	0.0024	0.30	0.0014	0.66	0.17	-	0.050	0.19	0.15	-	0.031	0.21
**AI**	0.022	-0.22	0.050	-0.19	0.31	-	0.28	-	0.30	-	0.28	-
***b*/*a***	<0.0001	-0.39	<0.0001	-0.39	0.23	-	0.34	-	0.21	-	0.31	-
***c*/*a***	0.034	-0.20	0.0013	-0.30	0.54	-	0.69	-	0.63	-	0.68	-
***d*/*a***	0.0033	0.28	0.11^a^	-	0.49	-	0.51	-	0.49	-	0.40	-
***e*/*a***	0.0004	0.34	0.0001	0.40	0.0007	0.32	0.001	0.31	0.0016	0.30	0.0003	0.34
***a*-*b***	0.036	-0.20	0.013	-0.24	0.68	-	0.50	-	0.50	-	0.47	-
***a*-*c***	0.058	-0.18	0.0027	-0.28	0.87	-	0.93	-	0.66	-	0.94	-
***a*-*d***	<0.0001	-0.53	<0.0001	-0.59	0.32	-	0.42	-	0.23	-	0.33	-
***a*-*e***	<0.0001	-0.62	<0.0001	-0.60	0.060	-	0.077	-	0.042	-0.19	0.029	-0.21

^a)^ Second order polynomial regression analysis, *p* = 0.044.

The only DPA variable that correlated with body height at Spearman’s rank correlation was *e*/*a*, but at linear regression analysis also DEI (Table 3).

In analogy with the strategy for adjusting the DPA variables for HR and/or body height, adjusted variable@75 values were compared with height in new regression analyses (Table 3). Then, Spearman’s rank correlation showed significant correlations for *e*/*a*@75 and (*a*-*e*)@75, and at linear regression also for DEI@75.

### Associations between DPA and tonometry variables

Table 4 shows the correlations between the DPA and tonometry variables. The crude and adjusted DPA variables showed correlations with both PWV and AIX@75, being statistically significant for all DPA variables except (*a*-*d*), (*a*-*d*)@75, and (*a*-*e*)@75@170, and for PWV also for (*a*-*e*).

**Table 4:** Correlation between tonometry parameters and DPA variables. Crude indicates unadjusted variables. @75 denotes adjustment of variables to a heart rate of 75 bpm.

DPA variables	Pulse wave velocity								Augmentation index @ heart rate 75							
	Crude				Adjusted				Crude				Adjusted			
	Spearman’s rank correlation		Simple linear regression		Spearman’s rank correlation		Simple linear regression		Spearman’s rank correlation		Simple linear regression		Spearman’s rank correlation		Simple linear regression	
	*p*	*rho*	*p*	*r*	*p*	*rho*	*p*	*r*	*p*	*rho*	*p*	*r*	*p*	*rho*	*p*	*r*
**EI**	< 0.0001	-0.55	< 0.0001	-0.48	< 0.0001	-0.57	< 0.0001	-0.53	< 0.0001	-0.63	< 0.0001	-0.62	< 0.0001	-0.64	< 0.0001	-0.66
**DI**	0.0002	0.36	<0.0001	0.37	0.0002	0.36	< 0.0001	0.42	0.0003	0.35	0.0002	0.35	0.001	0.32	0.0002	0.35
**DDI**	0.0004	-0.35	<0.0001	-0.36	0.0001	-0.37	<0.0001	-0.41	0.0003	-0.35	0.0002	-0.35	0.0002	-0.36	< 0.0001	-0.38
**EEI**	< 0.0001	-0.63	<0.0001	-0.56	< 0.0001	-0.66	<0.0001	-0.61	< 0.0001	-0.75	< 0.0001	-0.71	< 0.0001	-0.77	< 0.0001	-0.76
**DEI**	< 0.0001	-0.72	<0.0001	-0.49	< 0.0001^a^	-0.70	<0.0001^a^	-0.50^a^	< 0.0001	-0.69	< 0.0001	-0.58	< 0.0001^a^	-0.59^a^	< 0.0001^a^	-0.56^a^
**AI**	< 0.0001	0.72	<0.0001	0.65	< 0.0001	0.70	<0.0001	0.64	< 0.0001	0.79	< 0.0001	0.78	< 0.0001	0.78	< 0.0001	0.77
***b*/*a***	< 0.0001	0.62	<0.0001	0.55	< 0.0001	0.63	<0.0001	0.56	< 0.0001	0.76	< 0.0001	0.75	< 0.0001	0.77	< 0.0001	0.75
***c*/*a***	< 0.0001	-0.47	<0.0001	-0.43	< 0.0001	-0.54	<0.0001	-0.48	< 0.0001	-0.47	< 0.0001	-0.45	< 0.0001	-0.54	< 0.0001	-0.52
***d*/*a***	< 0.0001	-0.68	<0.0001	-0.60	< 0.0001	-0.68	<0.0001	-0.60	< 0.0001	-0.72	< 0.0001	-0.68	< 0.0001	-0.72	< 0.0001	-0.68
***e*/*a***	< 0.0001	-0.60	<0.0001	-0.50	< 0.0001^a^	-0.58	<0.0001^a^	-0.54^a^	< 0.0001	-0.67	< 0.0001	-0.63	< 0.0001^a^	-0.60^a^	< 0.0001^a^	-0.59^a^
***a*-*b***	0.0014	0.31	0.025	0.21	0.0027	0.29	0.034	0.20	0.046	0.19	0.0030	0.28	0.062	0.18	0.0065	0.26
***a*-*c***	0.13	-	0.025	-0.21	0.035	-0.20	0.0098	-0.25	0.0016	-0.30	0.0004	-0.33	0.0003	-0.35	< 0.0001	-0.38
***a*-*d***	0.90	-	0.41	-	0.21	-	0.11	-	0.58	-	0.85	-	0.51	-	0.46	-
***a*-*e***	0.31	-	0.80	-	0.50^a^	-	0.34^a^	-	0.015	-	0.084	-	1.0^a^	-	0.52^a^	-

^a)^ DPA variable adjusted for both heart rate and body height.

Adjustments for HR/height of the DPA variables resulted, with exceptions for AI@75 and (*a*-*b*)@75 related to PWV, and for DEI@75@170, AI@75, *e*/*a*@75@170 and (*a*-*b*)@75 related to AIX@75, in higher Pearson correlation coefficients, but in no instance did the coefficient increase by more than 0.05. There were minor changes in the levels of significance, but for (*a*-*e*) adjusted to (*a*-*e*)@75 the significant correlation with AIX@75 disappeared. Overall, the linear correlations were stronger for AIX@75.

### Agreement between ETc and LVET@60

**Fig 3:**
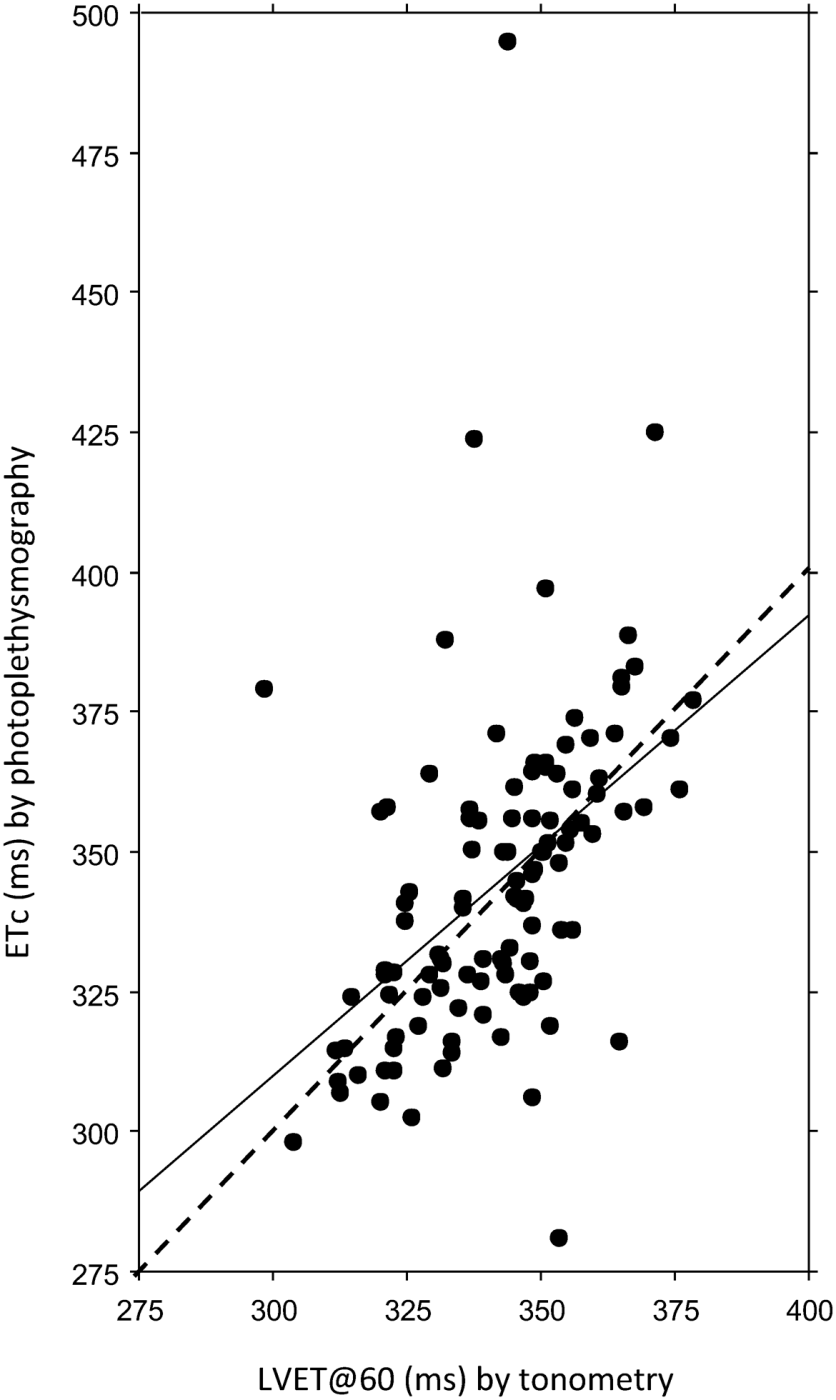
Association between ETc and LVET@60. Simple linear regression analysis of ejection time compensated (ETc) measured by photoplethysmography, and left ventricular ejection time adjusted to a heart rate of 60 bpm (LVET@60) measured by tonometry (r 0.46). The solid line represents the regression line and the interrupted line the line of identity.

The tonometry variable LVET was significantly and negatively correlated with HR (Spearman’s rho -0.74, *p* < 0.0001; simple linear regression r -0.81, *p* < 0.0001) but not with body height (*p* = 0.16 and 0.25, respectively); to compare the DPA variable ETc and the tonometer variable LVET, LVET was adjusted to a HR of 60 bpm, denoted LVET@60. A significant correlation was found between ETc and LVET@60 (Spearman’s rho 0.57, *p* < 0.0001; simple linear regression analysis r 0.46, *p* < 0.0001) (Fig 3).

**Fig 4:**
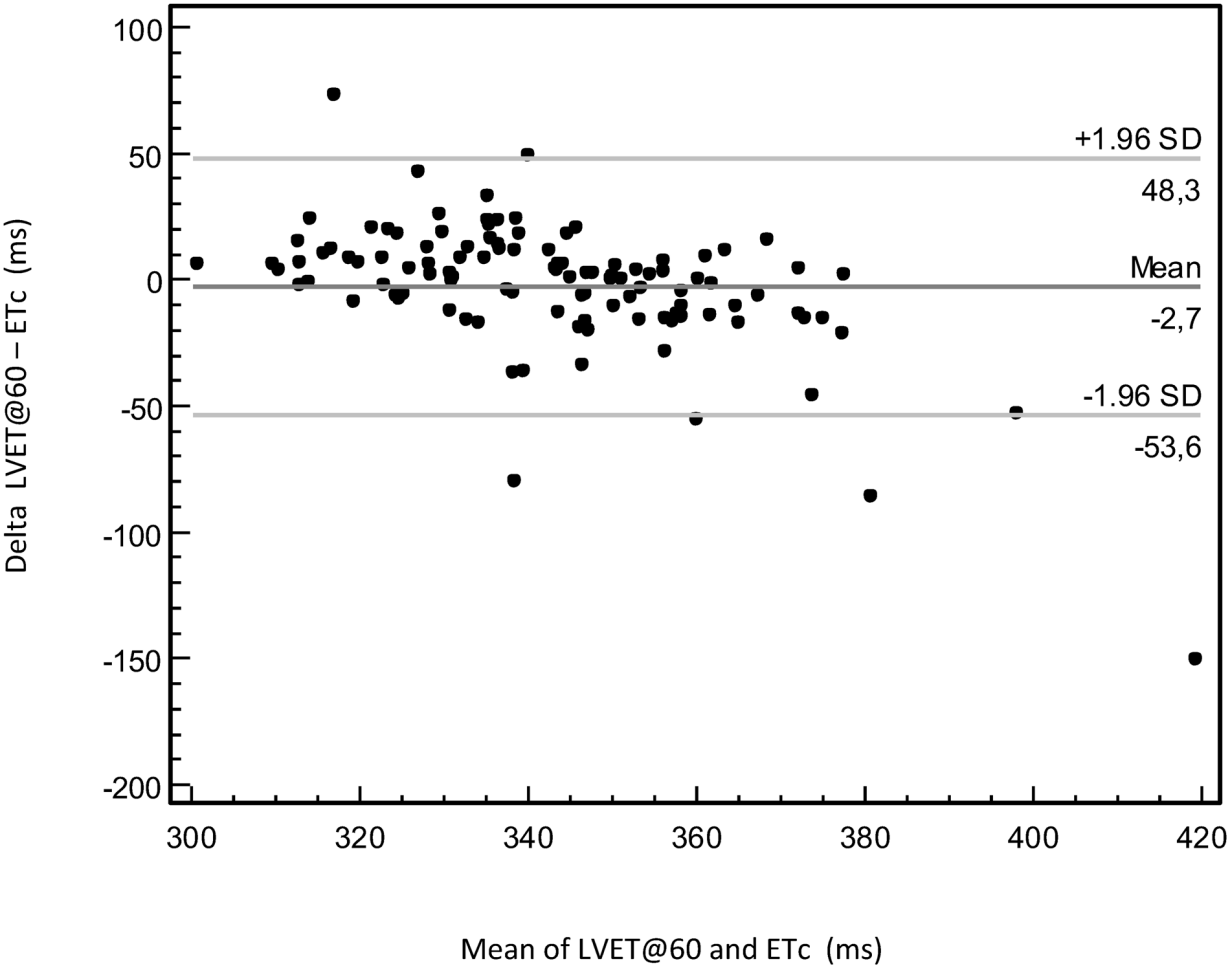
Agreement between ETc and LVET@60. Bland-Altman plot of left ventricular ejection time adjusted to a heart rate of 60 bpm (LVET@60) by tonometry and left ventricular ejection time compensated (ETc) by photoplethysmography, depicting the relationship of difference of measurements (Delta LVET@60 –ETc) and mean value of measurements.

A Bland-Altman plot (Fig 4) revealed a low overall bias in systolic ET between the methods (mean difference -2.7 ms); in the measurement range below about 340 ms the tonometer measurements of LVET tended to show slightly higher values than the DPA measurements of ETc. The ICC was 0.40.

## Discussion

Considering the levels of repeatability and the correlations to tonometry, the overall best DPA parameters were EEI, AI and *b*/*a*. The two pansystolic time parameters, ejection time compensated (ETc) by DPA and LVET by tonometry, showed a significant but weak correlation. For estimation of the LV function, ETc, EEI and *b*/*a* are suitable, for large artery stiffness EEI, and for small arteries DI and DDI. The only global parameter, AI, showed a high repeatability and the overall best correlations with AIX and PWV.

We deliberately chose to perform the study in a heterogeneous population sample, comprising both men and women and both pregnant and non-pregnant women of different ages. Such a population sample is expected to cover most components of the arterial stiffness concept, i.e. stiffness due to increased vascular tone, endothelial dysfunction, and structural elastic changes in the arterial wall. On the other hand, differences in age and gender have impacts on the accuracy of the pulse contour analyses [40], indicating that in a heterogeneous population like ours the repeatability of measurements differs from a homogenous population [34,41]. The ICC provides information of reliability of the parameters in relation to each other, where in the present study the ICC was poor for ETc, DEI, *a*-*c* and *a*-*d*, and strong for EEI, AI, *b*/*a* and *e*/*a*, but comparing parameter ICCs between studies is not meaningful unless the studies have equal population heterogeneity [34]. As illustrated by Bartlett & Frost [34], a measurement method could show a lower ICC in a homogenous population.

The agreement analyses were performed with Bland-Altman plots, which reflects the analysis method or instrument precision [34]. We found no general skewness for any DPA variable along the range of measurements. The repeatability indices are sensitive to outlying values [36] and we found for several DPA parameters that most outlying values were recorded from the same three persons. The characteristics of these persons were old age in two, low HR in two, and low PH in one. Most influential outliers were found for DEI, AI, *c*/*a* and *d*/*a*. After exclusion of the three persons from analyses, the CoRs for these parameters improved from 0.40–0.54 to 0.17–0.34.

As emphasized by Bland & Altman [36], repeatability indices must be evaluated in the clinical context and data obtained in a heterogeneous population may not be valid for other populations. It is then not meaningful to compare the absolute figures in the present study with figures obtained in other studies. We consciously disregarded possible morbidities and medications in the study group, because in this study we were interested in the DPA parameter performance from a repeatability perspective and not in whether the parameters reflected demographic and clinical characteristics.

The parameters ETc, EI, EEI and *b*/*a* represent in varying grades the LV ejection capacity, the parameters EI, EEI and *b*/*a* the stiffness in large arteries, and the parameters DI, DDI, DEI, *d*/*a* and probably also *a*-*d* the stiffness in small arteries and arteriole. AI, aging index, is a general arterial stiffness parameter. To select representative parameters with the best repeatability of the different vascular segments along the cardiovascular system, without discussing their functionality and clinical relevance, we judge that for the LV ejection capacity and large arteries they are EEI and *b*/*a*, and for small arteries it is DI and DDI that had the best reliability figures, i.e. highest ICCs. Also the reliability of AI was high (ICC 0.84), but for the only parameter representing the whole systole, ETc, it was poor (ICC 0.46).

The repeatability was generally stronger for DPA parameters representing the early systolic phase than for indices recorded in late systole or diastole. A common feature of three of the four variables with poor reliability in the present study, i.e. ETc, *a*-*c* and *a*-*d*, is that they all represent time axis measurement points occurring during late systole (*c* and *d* waves) or end of systole (point C). The *c*, *d* and *e* waves may not always be salient or exist as they are smoothed or merged, and false positive waves may occur due to high signal noise [42], which questions the robustness of variables relying on the identification of these inflection points. Also some DPA variables showing good reliability (DI, DDI) or moderate reliability (*c*/*a*, *d*/*a*) contain these inflections points, but in contrast to the other variables these variables represent quotas, which may be less sensitive to inexact positioning along the time axis.

The PPG signal is distorted and contaminated by noise from electrical circuits of the equipment and a correct identification of the APG waves is essential for calculation of wave peaks and wave crest time intervals, but there are few studies focusing on the automatic detection of these waves [42,43]. The APG wave detections are based on complex mathematical algorithms, including filtering of noise, second derivative of the filtered PPG signal, and demarcation of the index wave segment of interest to ignore signals from adjacent waves [42,43]. Filtering inevitably leads to distortion of time-related pulse features, such as arrival time of the reflected wave [44]. The DPA volume pulse is more damped than the tonometry pressure pulse because it is recorded in more peripheral vessels [45], suggesting that there might be a higher degree of uncertainty in the identification of PW contour inflections points of the DPA recordings.

There are no standard PPG databases available to evaluate different APG algorithms [43]; Elgendi et al. [43] tested nine different *a* and *b* wave detection algorithms in a database of recordings and found large differences between the algorithms to correctly detect the waves. Detection of the *c*, *d* and *e* waves is even more elusive [42]. The drawback that the signal processing is complex and sensitive to errors might be the reason why the DPA method has not been widely used in cardiovascular research [46].

The tonometric variables are influenced by changes in HR, though there is confusion whether HR should be controlled for or not [20]. In the present study, all DPA variables were significantly associated with HR, and DEI and *e*/*a* also with body height. Although the associations were highly significant, they were for most variables weak and for no variable did Spearman’s rho or Pearson’s correlation coefficient exceed 0.66. Regardless, in the comparisons with the tonometric variables the DPA variables were accordingly adjusted for differences in HR and body height. However, the adjustments resulted in only minor differences in significance levels and Pearson correlation coefficients compared to unadjusted DPA values. The question whether the DPA variables need to be adjusted for HR should be evaluated in each situation, and especially in situations where interventions influence the HR.

All DPA variables except the *a*-*d* and *a*-*e* variables were significantly associated with both AIX@75 and PVW, but the associations with AIX@75 were in general stronger. The strongest correlations were found for EEI, AI, and *b*/*a* (r 0.75–0.78); notably, all these variables contain *b*/*a*, representing the rising systolic slope of the PPG. An inverse relation exists between myocardial contractility and arterial compliance [47] and both the *b*/*a* ratio and the AIX reflect these entities. The strongest correlations with PWV were found for EEI, AI, and *d*/*a* (r 0.61–0.65). Weak to moderate correlations with PWV have been shown in other studies [16,48] and were expected, because none of the DPA parameters contain any modality alike the forward PW velocity along aorta. For example, an increase in acceleration of the early LV ejection phase would affect the *b*/*a* ratio but would not be detected as a change in PWV [45]. Hashimoto and coworkers [16] write that the weak correlations with PWV are not due to poor reproducibility of the PWV and DPA parameters, but occur because the parameters reflect different physiological properties at central and peripheral sites in the arterial vascular system. Hence, they cannot act as surrogate markers of each other.

No Pearson correlation coefficient was outstanding, but among the DPA variables it was AI that showed the strongest correlations with both AIX@75 and PWV (r 0.78 and 0.65, respectively).

With exception for the ETc and LVET, the DPA and tonometer variables are not directly interchangeable since they represent different technical methodologies measuring different physiological circulatory modalities. While the PWV is essentially reflecting the distensibility of the aortic wall and depends passively on arterial pressure, the DPA parameters depend on both central and peripheral vascular properties [45]. Moreover, factors like stress, temperature, hydration, etc., are more prone to affect the peripheral circulation than the central circulation. However, Millasseau et al. [12] found with simultaneous DPA and pressure PW recordings that the digital volume pulse is related to the peripheral artery pressure pulse by a constant transfer function over a wide range of influences.

It was outside the scope of the study to relate the DPA variables to clinical conditions, but the associations of AI, *b*/*a* and *d*/*a* with arterial stiffness and cardiovascular risk are well established in the literature [4,14–18].

Among the DPA and tonometer parameters, only the systolic time parameters ETc by DPA and LVET by tonometry directly correspond to each other. Already in 1971, Chirife et al. [49] stated that finger plethysmography is a reliable method to measure the ET. However, in our study the association between ETc and LVET was weak (rho 0.57, r 0.46). This is at least partly due to the fairly low reliability of the ETc measurements (ICC 0.46). To determine the systolic time, both the DPA and the tonometer methods utilize derivative mathematical remodellings of the crude PW curve contour to identify the time position of the closure of the aortic valves, i.e. the dicrotic notch. A correct identification of the dicrotic notch is therefore essential to determine the systolic time [27], but due to variations in HR, cardiac output and vascular tone, with drifting in time positioning of the dicrotic notch with distance from the aortic valve, and to interference with the reflected wave, its applicability has been questioned [30,46].

An additional uncertainty in the ET comparison is the corrections for differences in HR. In the Meridian DPA apparatus the ETc is automatically HR-adjusted by Bazett’s equation [32], originally developed to correct the ECG QT interval for HR, but we chose to adjust the LVET by the obtained LVET-versus-HR linear regression equation because Ishikawa et al. [50] found, in contrast to Jang et al. [39], that Bazett’s equation is not suitable for correction of LVET. The Bazett equation has been criticized for over-correcting at high HRs and under-correcting at low HRs [51].

We performed multiple statistical analyses including numerous PW variables, exposing the study to a risk of a type I error, but we consider that risk insignificant because the study showed consistent and no conflicting results.

In summary, this study was performed in a heterogeneous population because we wanted to test the limits of repeatability of a commercially available DPA. The results should be interpreted from that perspective. No DPA variable showed excellent repeatability; a good repeatability was found for DI, DDI, EEI, AI, *b*/*a* and *e*/*a*. No DPA variable showed systematic errors or skewness along the range of measurements. Almost all DPA variables were HR dependent, whereas almost none correlated with body height. In the comparisons with tonometry variables, adjustments of DPA variables for HR and height made no difference in the interpretation of the associations between parameters. The overall highest correlations were found with AIX, where EEI, AI and *b*/*a* were the only parameters showing a Pearson correlation coefficient >0.70; with PWV, a coefficient >0.60 was found only for EEI, AI and *d*/*a*. Considering the level of repeatability and the correlation with the established parameters for PW analysis, the best overall DPA parameters were EEI, AI and *b*/*a*. The only parameters defined equally by both equipments were ETc by DPA and LVET by tonometry. The correlation between these parameters was significant but fairly weak, which probably much depends on the poor repeatability of ETc, which in turn might be due to methodological difficulties to identify the end of systole. The DPA apparatus we used contained advanced mathematical algorithms to distinguish between LV performance, large artery stiffness and small artery stiffness; thus, for estimation of the LV function we regard ETc, EEI and *b*/*a* to be suitable, for large arteries EEI, and for small arteries DI and DDI. The only global parameter, AI, showed a high repeatability and the overall best correlations with the AIX and PWV. Although no DPA variable is a surrogate for applanation tonometry variables AIX and PWV, our validation of the DPA against the gold standard method suggests that DPA is a valuable tool for estimation of vascular status. Since the DPA method is fast, relatively inexpensive and operator independent, it could implicate advantages in clinical practice and screening.
